# High-throughput single-molecule mapping links subtelomeric variants and long-range haplotypes with specific telomeres

**DOI:** 10.1093/nar/gkx017

**Published:** 2017-02-09

**Authors:** Eleanor Young, Steven Pastor, Ramakrishnan Rajagopalan, Jennifer McCaffrey, Justin Sibert, Angel C.Y. Mak, Pui-Yan Kwok, Harold Riethman, Ming Xiao

**Affiliations:** 1 Drexel University, School of Biomedical Engineering, Philadelphia, PA, 19104 USA; 2 Cardiovascular Research Institute, University of California, San Francisco, CA, 94158 USA; 3 Old Dominion University, Medical Diagnostic and Translational Sciences, Norfolk, VA, 23529 USA; 4 Institute of Molecular Medicine and Infectious Disease, School of Medicine, Drexel University, Philadelphia, PA, 19102 USA

## Abstract

Accurate maps and DNA sequences for human subtelomere regions, along with detailed knowledge of subtelomere variation and long-range telomere-terminal haplotypes in individuals, are critical for understanding telomere function and its roles in human biology. Here, we use a highly automated whole genome mapping technology in nano-channel arrays to analyze large terminal human chromosome segments extending from chromosome-specific subtelomere sequences through subtelomeric repeat regions to terminal (TTAGGG)n repeat tracts. We establish detailed maps for subtelomere gap regions in the human reference sequence, detect many new large subtelomeric variants and demonstrate the feasibility of long-range haplotyping through segmentally duplicated subtelomere regions. These features make the method a uniquely valuable new tool for improving the quality of genome assemblies in complex DNA regions. Based on single molecule mapping of telomere-terminal DNA fragments, we provide proof of principle for a novel method to estimate telomere lengths linked to distinguishable telomeric haplotypes; this single-telomere genotyping method may ultimately enable delineation of human cis elements involved in telomere length regulation.

## INTRODUCTION

Telomere-adjacent DNA is crucial for telomere (TTAGGG)n tract length regulation and telomere integrity. The non-coding telomeric repeat-containing RNA (TERRA) is transcribed from the subtelomere into the (TTAGGG)n tract (1–3) and forms an integral component of a functional telomere; perturbation of its abundance and/or localization causes telomere dysfunction and genome instability (1,4). DNA elements cis to the (TTAGGG)n tract regulate both TERRA levels and haplotype-specific (TTAGGG)n tract length and stability (4–8), with accumulating evidence for specific epigenetic modulation of these effects (8–11). More extended subtelomere regions contain both coding and non-coding transcripts, the abundance and regulation of which are likely to depend upon the specific haplotypes and copy number of the DNA encoding them. While some of these transcripts are clearly functional, most are not well-characterized (12–16). Large structural variations in subtelomere DNA exist, and the altered juxtaposition of subtelomeric sequence elements and 1-copy DNA relative to the telomere may affect gene expression and the packaging of telomeric chromatin. *De novo* deletion of subtelomeric duplications can cause disease in some contexts (17), and long-range interactions of telomeres with subtelomeric genes can regulate the expression of specific subtelomeric genes in a telomere length-dependent fashion (18,19). Accurate maps and DNA sequences for human subtelomere regions, along with detailed knowledge of subtelomere variation and long-range telomere-terminal haplotypes in individuals, are critical for understanding telomere function and its roles in human biology. The distal 500 kb of each chromosome arm encompasses all known multi-telomere duplications and is defined as the ‘subtelomere’ for our purposes.

Genomic regions of high segmental duplication content and/or structural variation have led to gaps and misassemblies in the human reference sequence. Ambiguities in sequence localization because of duplication content, as well as the presence of alternative haplotypes differing by relatively large insertions, deletions and more complex sequence organization differences, contribute to these gaps and misassemblies. Human subtelomere regions are enriched in both segmental duplication content and structural variations. In spite of these complications, specific individual haplotypes of subtelomere/telomere regions of human chromosomes were successfully mapped, sequenced and incorporated into the human reference sequence by (i) using specialized yeast artificial chromosome (YAC) clones spanning subtelomeric segmental duplication regions to connect terminal (TTAGGG)n tracts with chromosome-specific sequences; the YAC clone-specific copies of the subtelomeric segmental duplications were sequenced individually (20); and (ii long-range PFGE-based mapping studies done to demonstrate physical proximity of BAC clone-based sequence assemblies to telomeres (16,21). While contributing substantially to the human reference sequence, these studies were labor-intensive and incomplete, leaving small gaps adjacent to some (TTAGGG)n tracts as well as entire alternative subtelomeric haplotypes detected but uncharacterized. Telomere clones from a fosmid structural variation resource (22) have been used to fill in relatively small (TTAGGG)n-adjacent sequence gaps (23), but the sequence content and organization of larger alternative subtelomeric haplotypes are largely unexplored. All of these studies were dependent on a few large-insert clone libraries, precluding their extension to a large number of genomes.

We have recently developed a highly automated whole genome mapping technology in nano-channel arrays (24,25), which has been applied in assisting *de novo* genome assembly and structural variation analysis (26,27). In this report, we use this novel, highly accurate, high-throughput, single-molecule mapping technique to analyze large terminal human chromosome segments extending from chromosome-specific subtelomere sequences through subtelomeric repeat regions to terminal (TTAGGG)n repeat tracts. We establish detailed maps for subtelomere gap regions in the human reference sequence, detect many novel large subtelomeric variants and demonstrate the feasibility of long-range haplotyping through segmentally duplicated subtelomere regions. Based on single molecule mapping data, we are also able to estimate telomere length for each mapped telomere. High-throughput single molecule mapping opens the door to the detailed characterization of difficult genomic regions using long uncloned single genomic DNA molecules.

## MATERIALS AND METHODS

### High molecular weight DNA extraction

Mammalian cells were embedded in gel plugs and high molecular weight DNA was purified as described in a commercial large DNA purification kit (BioRad #170-3592). Plugs were incubated with lysis buffer and proteinase K for 4 h at 50°C. The plugs were washed and then solubilized with GELase (Epicentre). The purified DNA was subjected to 4 h of drop-dialysis. It was quantified using Quant-iTdsDNA Assay Kit (Life Technology), and the quality was assessed using pulsed-field gel electrophoresis.

### DNA labeling

The DNA was labeled with nick-labeling (28) as described previously using the IrysPrep Reagent Kit (BioNano Genomics). Specifically, 300 ng of purified genomic DNA was nicked with 7 U nicking endonuclease Nt.BspQI (New England BioLabs, NEB) at 37°C for 2 h in NEB Buffer 3.1. The nicked DNA was labeled with a fluorescent-dUTP nucleotide analog using Taq polymerase (NEB) for 1 h at 72°C. After labeling, the nicks were ligated with Taq ligase (NEB) in the presence of dNTPs. The backbone of fluorescently labeled DNA was stained with YOYO-1 (Invitrogen).

### Data collection

The DNA was loaded onto the nano-channel array of BioNano Genomics IrysChip by electrophoresis of DNA. Linearized DNA molecules were imaged using a custom made whole genome mapping system. The DNA backbone (outlined by YOYO-1 staining) and locations of fluorescent labels along each molecule were detected using an in-house image detection software. The set of label locations relative to the DNA backbone for each DNA molecule defines an individual single-molecule map. A commercial version of this whole-genome mapping and imaging system (Irys) is available from Bionano Genomics.

### *De novo* genome map assembly

Single-molecule maps were assembled *de novo* into consensus maps using software tools developed at BioNano Genomics, specifically Refaligner and Assembler (26). Briefly, the assembler is a custom implementation of the overlap-layout-consensus paradigm with a maximum likelihood model. An overlap graph was generated based on pairwise comparison of all molecules as input. Redundant and spurious edges were removed. The assembler outputs the longest path in the graph and consensus maps were derived. Consensus maps are further refined by mapping single molecule maps to the consensus maps and label positions are recalculated. Refined consensus maps are extended by mapping single molecules to the ends of the consensus and calculating label positions beyond the initial maps. After merging of overlapping maps, a final set of consensus maps was output and used for subsequent analysis. The map assemblies are very robust to the relatively small errors in labeling (10% false positive, due to extra nickings at wrong sites and 10% false negative, due to missing nicks). This does not affect the maps and haplotype calls as the haplotypes are both are based on multiple nicking sites and multiple single molecules.

### Structural variation detection

Structural variants (SVs) were found by identifying outlier alignments between single-molecule maps/genome maps from a sample and the reference maps (26).

### Telomere length estimate

Single DNA molecules aligned to the consensus map in the subtelomeric regions were used for the telomere length measurements. We specifically selected molecules that were long enough to extend into 1-copy subtelomere regions in order to ensure the uniqueness of the alignment and localization to a specific subtelomeric region. We excluded any molecule with an alignment confidence score <20 based on Realigner's results (26). The strategy of estimating the telomere length through single DNA molecule mapping is based on the following facts: (i) the origin of the reference sequence starts from the beginning of subtelomeric sequences (p-arm) and the end of the reference sequence stops at the end of subtelomeric sequences (q-arm). (ii) The reference sequences lack significant stretches of (TTAGGG)n repeat (16,23). (iii) There is no nicking motif in the telomere repeats. In estimating the telomere length, we first determine the first or last nicking site of a single DNA molecule that is aligned to the human reference genome (p-arm or q-arm respectively), then we calculate the extra length of the DNA molecules beyond the origin or the end of the reference sequence; this length corresponds to telomere repeats (Figure 5). While clearly more expensive at its current stage of development than conventional methods for measuring average telomere lengths, this new method provides qualitatively different datasets that link single telomere lengths with specific subtelomeres and subtelomere haplotypes; the proof of principle we demonstrate for this novel single-telomere genotyping method may ultimately enable delineation of previously inaccessible human subtelomeric cis elements involved in telomere length regulation.

## RESULTS

### Individual subtelomeric consensus maps containing large SRE regions

Subtelomeric repeat element (SRE) regions are located in the most distal stretches of human subtelomeres. Long SRE regions of about 300 kb have been identified in some alleles of the 1p, 8p and 11p telomeres, whereas 7 telomeres have minimal or no SRE content (16,20,23,29). Most SRE regions are 40–150 kb in size (23). Physical linkage of 1-copy regions with telomeres on single large DNA molecules capable of spanning SRE regions is required for assembling individual subtelomeric consensus maps. Read lengths of >50–300 kb would be required for assembling these regions using single-molecule sequencing, which is beyond the capability of current technology (30). However, recently-developed high-throughput single-molecule genome mapping methods may be well-suited for this challenge. In this method, genomic DNA is labeled at sites recognized by a sequence motif-specific Nicking endonuclease, long genomic DNA fragments are isolated and imaged in nanochannel arrays to a high depth of coverage and contigs of these large genomic DNA fragments are assembled from these data. In our case, these maps are then compared with *in silico*-generated maps of subtelomeric reference sequences.

**Figure 1.**
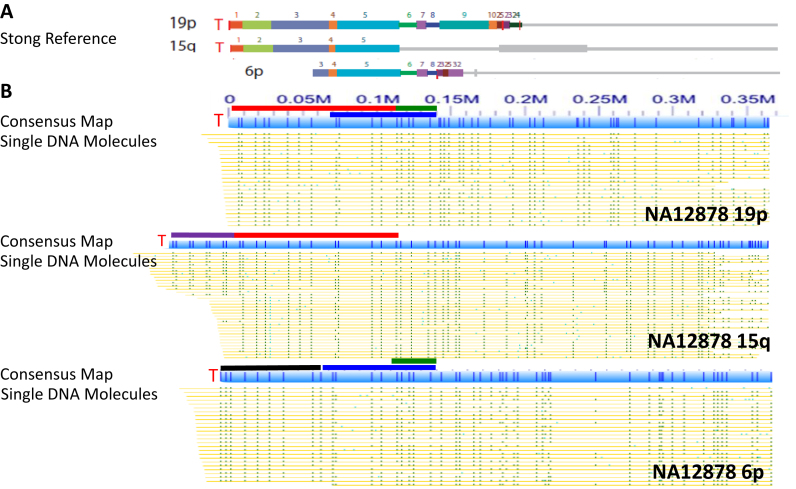
Consensus genome maps of individual subtelomeric regions containing SRE regions of NA12878. (**A**) Human subtelomere reference assemblies as described in Stong *et al.* (23) for 19p, 15q and 6p. Subtelomeric Repeat Element (SRE) paralogy blocks are shown in colors, with the gray line segments indicating adjacent 1-copy subtelomere DNA. The 19p and 15q assemblies extend to the start of the telomere terminal repeat (TTAGGG)n tract on the left (T), whereas there is a gap of indeterminate size between the start of the 6p reference sequence and the telomere. (**B**) Consensus single-molecule maps from the NA12878 genome aligning to the 19p, 15q and 6p telomeres of the Stong *et al.* (23) reference assemblies. In the consensus map for each subtelomere, nicking sites are represented by dark blue vertical lines within the light blue rectangles above the single DNA molecule maps. Each yellow row represents a single mapped large genomic DNA molecule, with the green ticks on each yellow row showing the labeled nicking sites imaged on that molecule. Lighter green ticks indicate nicking sites that did not match to the reference sequence. The numbers above the 19p consensus map indicate distance in megabases (Mb). The red bars in Panel B show SRE paralogy region blocks 1–5 from panel A that are shared by 19p and 15q. The dark blue bars in Panel B show SRE paralogy region blocks 4–8 from Panel A that are shared between 19p and 6p as well as part of 15q. The green bars in Panel B show a DNA segment shared between 19p and 6p but not 15q. The purple bar in Panel B shows a structurally variant segment of the 15q subtelomere, an insertion of about 50 kb immediately adjacent to the telomere relative to the reference sequence. The black bar in Panel B indicates the 6p gap region delineated by the consensus map.

Figure 1 shows the consensus maps of the 19p, 15q and 6p subtelomeres from the NA12878 genome, aligned with the subtelomere assemblies of Stong *et al.* (23). The human 19p, 15q and 6p subtelomeres share some SREs but differ in others, as shown in Figure 1A. 19p and 15q share duplicons 1–5 of 120 kb. 6p, which contains a gap between the telomere and the beginning of the reference sequence, shares a partial duplicon 3 as well as duplicons 4–8 with 19p (Figure 1A). 19p, 15q and 6p of NA12878 were *de novo* assembled into unique consensus maps from the mixture of single long DNA molecules (Figure 1B). 15q of NA12878 has two different haplotypes, and one of the haplotypes has extra telomere-adjacent subtelomeric sequences compared to the reference (the purple bars in Figure 1B). However, both haplotypes of 15q share the same patterns of about 120 kb with 19p (the red bars of Figure 1B), which are nearly identical (98–99.5% similarity) in the reference sequences of 19p and 15q from 1 to 120 kb. As indicated in the blue bars in Figure 1B, the consensus maps of 19p and 6p support that they share duplicons 4–8. The subtle difference of lacking duplicons 6–8 in 15q, compared to 19p and 6p, is also demonstrated in Figure 1B (green bars from 120 to 140 kb). Since all the single molecules used in the consensus maps in Figure 1 are longer than 300 kb, and contain 1 copy unique segments centromeric to the SREs (gray bars in Figure 1A), this mapping method can clearly distinguish individual SRE regions within a genome. Thus, this genome mapping method with long DNA molecules provides a unique tool to track and study individual SRE variants from within families of SREs by their physical linkage to 1-copy DNA on single molecules.

### Discovery of novel subtelomeric structural variants, resolution of sequence gaps and delineation of long-range subtelomeric haplotypes

**Figure 2.**
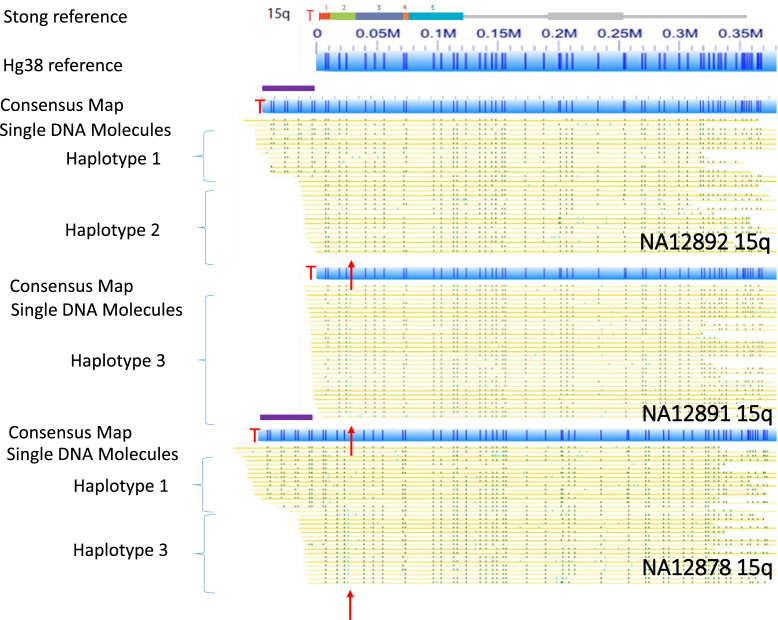
Haplotype-resolved subtelomeric variants of 15q. SRE paralogy blocks from Stong *et al.* reference assembly (23) are shown in colors, with the gray line segments indicating adjacent 1-copy subtelomere DNA. The 15q assembly begins at the centromeric end of the telomere terminal repeat (TTAGGG)n tract on the left (T). The hg38 representation of this assembly, which is identical to that of Stong *et al.* (23) except for the addition of a 10 kb gap adjacent to the telomere to represent unsequenced (TTAGGG)n (see supplementary materials), has been in silico nicked with the Nicking enzyme Nt.BspQI used for genome-wide mapping (represented by dark blue vertical lines within the light blue rectangles). Consensus single-molecule maps from the NA12892, 12891 and 12878 genomes aligning to the 15q telomeres are represented as described in Figure 1, and positioned beneath the hg38 reference map. Each yellow row represents a single molecule, with nicking sites shown in green. NA12892 contains haplotypes 1 and 2, while NA12891 has a third haplotype and NA12878 has haplotypes 1 and 3. The red arrows indicate the position of a nicking site that is present in the third haplotype but absent in the first and second haplotypes.

**Figure 3.**
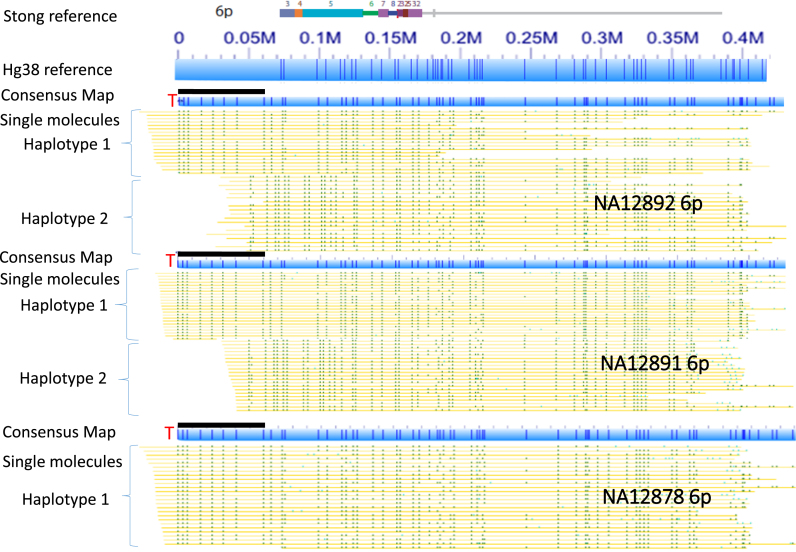
Gap characterization and subtelomeric haplotype structures of 6p. SRE paralogy blocks from Stong *et al.* (23) are shown in colors, with the gray line segments indicating adjacent 1-copy subtelomere DNA. The 6p subtelomere assembly contains a gap of indeterminate size between the telomere and the start of the assembly. This gap is omitted in the Stong *et al.* (23) reference, and represented in the hg38 reference by a telomere (TTAGGG)n gap (10 kb) plus a clone gap (50 kb) on the telomeric side of the 6p assembly start position (see Supplementary Data); the hg38 reference is otherwise identical to the Stong *et al.* (23) reference sequence. *In silico* nicking of the hg38 reference with the Nicking enzyme Nt.BspQI used for genome-wide mapping is shown (represented by dark blue vertical lines within the light blue rectangles); note that the subtelomere gap region of hg38, a 60 kb string of NNN's in the sequence itself, has no nicking sites represented in the electronic digest. Consensus single-molecule maps from the NA12892, 12891 and 12878 genomes aligning to the 6p telomere are represented as described in Figure 1, and positioned beneath the hg38 reference map. The position of the gap region of the hg38 reference sequence is represented above each consensus map by a black rectangle. NA12892 shows two structurally variant haplotypes, as does NA12891, but NA12878 has inherited only haplotype 1 from each parent. While structurally variant within the gap region of 6p, the two haplotypes clearly diverge ∼120 kb from the telomere within SRE paralogy region 5.

Encouraged by our initial results with the NA12878 genome, we next analyzed in-depth the mapping data from NA12878 as well as data acquired in a similar fashion from five additional genomes, two of which form a family trio with NA12878. Figure 2 shows single-molecule consensus maps of the 15q subtelomeric region in genomic DNA from a maternal grandmother (NA12892), maternal grandfather (NA12891) and mother (NA12878) family trio from the CEPH collection. Based on the alignment of these maps with the reference sequence, NA12892 and NA12878 are heterozygous, while NA12891 is homozygous. NA12892 and NA12878 share the same SV (haplotype 1), which has about 50 kb more DNA (purple bars in Figure 2) than the completed 15q reference sequence. The consensus maps of NA12892 (haplotype 2) and NA12878 (haplotype 3) are similar, but haplotype 2 of NA12892 is missing a nicking site (green ticks indicated by red arrow in Figure 2). The homozygous NA12891 has the same consensus map as haplotype 3. Clearly, NA12878 inherited the longer haplotype 1 from NA12892, and the shorter haplotype 3 from NA12891. The molecules shown in Figure 2 are all longer than 300 kb, and extend into the 1- copy unique region of the 15q subtelomere. This excludes the possibility of molecules from different chromosomes but with similar duplicon structures obfuscating the consensus maps. Figure 3 shows long-range subtelomeric haplotypes of 6p from the same CEPH trio. In this case, two haplotype-resolved consensus maps are confirmed, and the 6p subtelomere gap region in the current reference sequence is shown to contain a large subtelomeric SV.

Many additional large subtelomeric SVs (over 20 kb), new gap-filling DNA regions and novel long-range subtelomeric haplotypes were found in the six genomes analyzed (Table 1). For 3q, all six genomes have the same consensus map with 130 kb telomere-adjacent sequences (Supplementary Figure S1) filling the gap in the reference sequence to the start of the 3q telomere sequence (23). Similarly, the consensus map of 11p fills an existing gap in the reference sequence with a defined mapping pattern for an additional 93 kb of DNA (Supplementary Figure S1), and a small amount of DNA is added to the gap region of 8p for all six genomes (data not shown). 7p has 91 kb of additional sequence with a defined mapping pattern for all six genomes; however, in this case the existing reference sequence defines a complete 7p, indicating a large, more common SV 7p allele in the 6 genomes (Supplementary Figure S1). The consensus maps of 9q and 20q show 25 and 56 kb of additional telomere-adjacent sequences beyond the current reference sequence, respectively (Supplementary Figure S1).

**Table 1.** Subtelomere gaps (g), haplotypes (h) and structural variants (sv) characterized

Tel	NA12878	NA12891	NA12892	HG02603	HG02490	HG02522	Large-scale variation^a^
1p	g	g, h, sv	g	nd	nd	nd	Hi
1q	-	-	-	-	-	-	Lo
2p	-	-	-	-	-	-	Lo
2q	-	-	-	-	-	-	Lo
3p	-	-	-	-	-	nd	Lo
3q	g	g	g	g	g	g	Lo
4p	-	-	-	-	-	-	Lo
4q	sv	sv	sv	sv	sv	sv	Hi
5p	-	-	-	-	-	-	Lo
5q	-	-	-	-	-	-	Lo
6p	g	g, h, sv	g, h, sv	g	nd	nd	Hi
6q	-	-	h				Lo
7p	sv	sv	sv	sv	sv	sv	Hi
7q	-	-	-	-	-	-	Lo
8p	g	g	g	g	g	g	Hi
8q	-	-	-	-	-	-	Lo
9p	h	h	h	-	-	-	Lo
9q	g	g	g	g	g	nd	Hi
10p	-	-	-	-	-	-	Lo
10q	h, sv	h, sv	h, sv	h, sv	sv	sv	Hi
11p	g	g, sv, h	g	nd	g	g	Hi
11q	-	-	-	-	-	-	Lo
12p	-	-	-	-	-	-	Hi
12q	-	-	-	-	-	-	Lo
13q	-	-	-	-	-	-	Lo
14q	sv	sv	sv	sv	sv	sv	Hi
15q	sv, h	h	sv	-	-	-	Lo
16p	nd	nd	nd	nd	nd	nd	Hi
16q	h	h	h	sv	-	sv	Hi
17p	nd	nd	nd	nd	nd	nd	Hi
17q	-	-	-	-	-	-	Lo
18p	-	-	-	-	-	-	Lo
18q	-	-	-	-	-	-	Lo
19p	nd	-	-	-	-	-	Hi
19q	nd	nd	nd	nd	nd	nd	Hi
20p	g	g	g	g, sv	g	g, sv	Hi
20q	sv	sv	sv	sv	sv	sv	Hi
21q	-	-	-	-	-	-	Lo
22q	nd	nd	nd	nd	nd	nd	nd
Xp/Yp	nd	nd	nd	nd	nd	nd	nd
Xq/Yq	-	-	-	-	nd	-	Lo

^a^Telomeres with a frequency of >10% large variant alleles in the small populations sampled are considered to have ‘Hi’ polymorphism in the context of this paper, and those with less than 10% large variant alleles are considered to have ‘Lo’ polymorphism. For the telomeres listed as ‘nd’, no molecular data are available with respect to large-scale variations and the available FISH data are inconclusive with respect to potential large-scale variation. The polymorphism frequencies detected by FISH are minimum numbers, since detection depends upon the variable presence/absence of only one specific FISH probe at the telomere. The size(s) of the polymorphisms cannot be determined by FISH, but are assumed to be at least the size of the probe used (based upon similar FISH signal intensities at all sites). Data on polymorphic telomeres are from: (21,29,31,32,39–45), Riethman, Unpublished results, and this paper.

g = gaps

h = haplotypes

sv = structural variants

‘-’ = no difference compared to Stong *et al.* (23) reference sequence

nd = no data

16q and 20p show variable lengths of telomere-adjacent sequences among different individuals (Supplementary Figure S2). The consensus maps of 16q of NA12892, NA12891,NA12878 and HG02490 are identical to the completed 16q reference sequence of (23), whereas 16q of HG02522 and HG02603 extend 110 kb beyond this reference sequence (Supplementary Figure S2A). The consensus maps of 20p show three variable lengths of telomere adjacent sequences from 98 to 238 kb between individuals (Supplementary Figure S2B), suggesting a very high level of structural variation at this subtelomere, as had been noted previously (29,31,32). The polymorphism of 14q consensus maps consists of multiple insertions (green bars in Supplementary Figure S3) and deletions (orange bars in Supplementary Figure S3) between different individuals, as might be expected from the variable IgG heavy chain locus which can become somatically rearranged in white blood cell clones immortalized during lymphoblastoid cell line generation.

Interestingly, the 4q consensus maps not only show the expected variable lengths of the subtelomeric macrosatellite D4Z4 array (33) among different individuals, but also have adjacent 35 kb deletions relative to the reference sequence for some individuals (Supplementary Figure S4). The 10q consensus maps also indicate variable lengths of the D4Z4 macrosatellite array among different individuals (Supplementary Figure S5), with HG02603 having two haplotypes, showing distinctly different lengths of D4Z4 tracts of 40 and 62 kb. In addition, the 10q consensus maps of the related NA12878, NA12891 and NA12892 genomes have a large 139 kb insertion (green bar) 400 kb upstream from the telomere, apparently unrelated to the D4Z4 repeat array.

### Internal structural variants in subtelomeric regions

**Figure 4.**
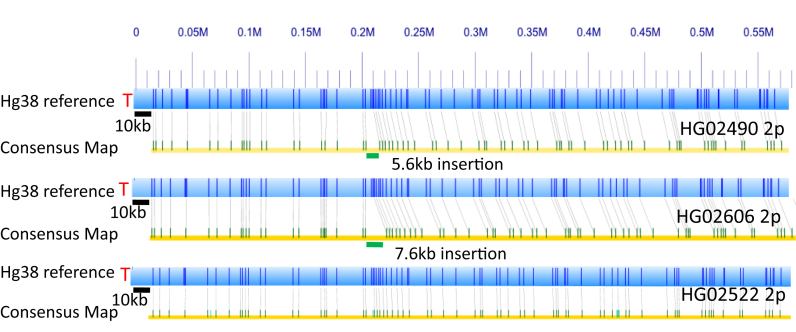
Detection of internal subtelomeric structural variations. The *in-silico* nicked 2p subtelomere region of the hg38 assembly is shown directly above consensus single-molecule maps of this region (represented by the yellow bar with green ticks) for each of the HG02490, HG02606 and HG02522 genomes. Note the 10 kb gap region represented at the start of hg38 (see Supplementary Data). Both HG02490 and HG2606 show an insertion at coordinate 0.2Mb. The blue bar here is the reference, hg38 and the yellow bar represents the consensus map of the molecules. Light gray lines connect the green dashes, which indicate nicking sites, to their locations on hg38. At 0.2Mb an insertion occurs on both HG02606 and HG02490.

Based on the single molecule assembled contigs, we assigned specific consensus maps to subtelomere haplotypes, resolved existing gaps in the subtelomere reference sequence and identified large SVs in the distal subtelomere regions. However, using the same six datasets, we were also able to identify new conventional SVs in more internal regions of the subtelomere maps. Typical examples of these are shown in Figure 4. For the six genomes studied in this report, the single molecule mapping evidence for internal subtelomere regions was consistent with the current reference sequences for 5q, 6q, 8q, 18q and 19p. However, we used our single-molecule maps and consensus maps to detect 33 insertion and 14 deletion loci located in internal subtelomeric regions (Supplementary Table S2) for HG02603, HG02490 and HG02522. All of these insertions and deletions were carefully verified by manual inspection of the raw single-molecule mapping data to confirm supporting evidence. The SVs of NA12878, NA12891 and NA12892 were reported previously (26).

### Telomere length estimate by single molecule DNA mapping

There is currently no good method for comprehensive molecular analysis of single telomere lengths in the human genome, which can quantify a critically important biological parameter currently masked in all experimentally scalable analyses of telomeres; the relative global fraction and identities of very short telomeres in a given sample of genomic DNA (34).

**Figure 5.**
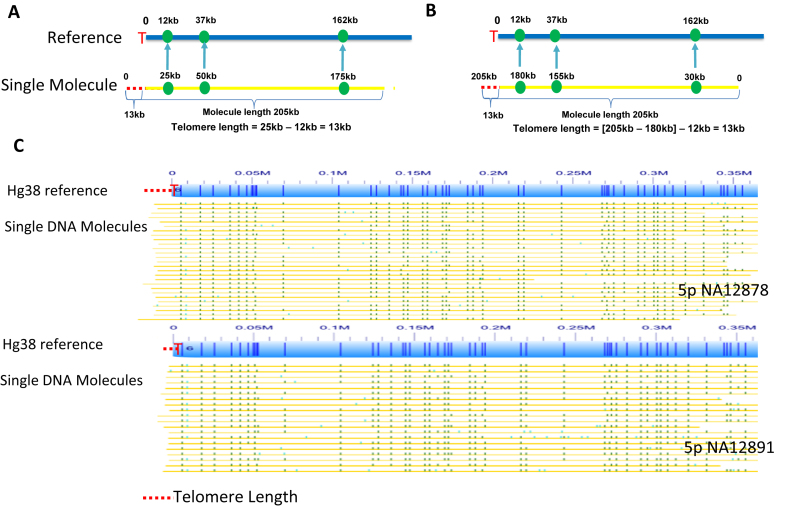
Telomere length measurement. The strategy of telomere length estimation through single-molecule mapping is shown in (**A** and **B**). Panel A shows a single DNA molecule (yellow line) that is aligned to the reference sequences (blue line). The telomere end (dotted red line) of the DNA molecule entered the nano-channel first. The three fluorescent labels (green dots on the yellow line) on the DNA molecule are mapped to the sequence motifs on the reference (green dots on the blue line). Panel B shows the same molecule that entered the nano-channel in a different orientation, with the telomere end entering the last. Panel **C** shows the single molecules used to estimate the telomere lengths from chromosome 5p of NA12878 and NA12891.

The ability to accurately map individual DNA molecules to discrete subtelomere regions provides a unique method of estimating telomere lengths, and ultimately connecting telomere length measurements with individual subtelomere haplotypes. Figure 5A and B show a schematic diagram of our single-molecule telomere length estimation strategy. It identifies the subtelomere of a single DNA molecule (yellow line) by aligning its sequence motif pattern (green dots) to the *in silico*-nicked subtelomere reference sequence (blue line). In Figure 5A, the single DNA molecule of 205 kb in length entered the nano-channel with the telomere end (red dotted line) first. The three fluorescent labels were localized from the telomere end (red dotted line) at 25, 50 and 175 kb, which were mapped to the reference at 12, 37 and 162 kb respectively. Since the subtelomere starts from 0 on the reference sequence and the first nicking motif at 25 kb of the DNA molecule matches to the reference at 12, 13 of 25 kb (25–12 kb) of the single molecule is the telomere (dotted red line). There should be no fluorescent labels of this 13 kb, except random false positive labels, as telomere repeats do not contain any nicking motifs. Figure 5B displays the same molecule entering the nano-chanel from the non-telomere end first. The three fluorescent labels were localized at 30, 155 and 180 kb, which were aligned to the reference at 162, 37 and 12 kb respectively. In this case, the telomere length is calculated to be 13(205-180-12 kb). In Figure 5C, many single molecules are aligned to the reference to estimate the single telomere lengths from chromosome 5p of NA12878 and NA12891. It is visually clear that NA12878 has the longer average telomere length than NA12891.

Table 2 summarizes the telomere length estimates of three samples in the NA12878 trio family. A total of 36 out of 46 possible telomeres were measured. Five acrocentric short-arm telomeres and five poorly mapped telomeres lack telomere length estimates. The haplotype 2 of 16q (NA12891) has the shortest average telomere length of 1.5 kb, while 4q of NA12892 has the longest average telomere length. It is important to note that the standard deviations reflect biological differences as well as measurement differences; since the measurements are made on single molecules, and the sample is a large collection of cells each having a different population doubling trajectory, true single-cell fluctuations in telomere lengths about a mean at single telomeres are expected (Baird *et al.* (35)). Standard deviations for molecular length measurement of similarly sized single molecules lacking telomeres using single-molecule mapping are typically ±250 bp (36).

**Table 2.** Telomere length estimate by single molecule measurement

	NA12878	NA12891	NA12892		NA12878	NA12891	NA12892
Chr-parm	Length in kb:Mean ± Std (number of telomeres)			Chr-qarm	Length in kb:Mean ± Std (number of telomeres)		
1p*	17.7 ± 3.9 (62)	12.7 ± 4.9 (14)	17.7 ± 6.4 (31)	1q	8.7 ± 5.3 (52)	4.5 ± 2.3 (21)	9.8 ± 5.1 (21)
2p	8.7 ± 3.7 (94)	2.8 ± 1.8 (21)	10.4 ± 3.9 (18)	2q	9.2 ± 4.0 (48)	4.7 ± 3.0 (13)	9.5 ± 4.0 (25)
3p	9.5 ± 3.8 (20)	5.2 ± 3.5 (11)	14.3± 7.7 (14)	3q*	14.5 ± 1.5 (3)	9.0 ± 1.7 (6)	19.7 ± 1.3 (3)
4p	8.8 ± 4.0 (34)	5.0 ± 3.7 (17)	16.5 ± 5.8 (19)	4q	nd	nd	34.4 ± 4.3 (2)
5p	10.6 ± 4.0 (24)	7.9 ± 4.9 (10)	11.7 ± 4.8 (13)	5q	8.7 ± 6.0 (81)	4.6 ± 3.7 (24)	8.4 ± 3.8 (17)
6p(haplo-type-1)*	14.8 ± 3.3 (29)	11.2 ± 1.3 (14)	19.0 ± 3.5 (18)	6q	3.6 ± 3.2 (47)	3.6 ± 2.8 (7)	4.8 ± 3.6 (20)
6p(haplo-type-2)*	no haplotype-2	10.7 ± 2.5 (10)	18.2 ± 4.5 (16)	7q	8.5 ± 4.0 (28)	3.2 ± 1.6 (37)	12.1 ± 4.9 (26)
7p*	16.0 ± 4.0 (13)	13.9 ± 5.75 (12)	18.8 ± 14.9 (12)	8q	9.4 ± 3.5 (56)	4.9 ± 1.9 (13)	11.6 ± 3.3 (22)
8p*	9.1 ± 4.0 (59)	3.1 ± 1.8 (23)	7.6 ± 4.3 (17)	9q*	12.4 ± 1.7 (31)	12.4 ± 1.7 (31)	10.4 ± 4.8 (15)
9p	9.6 ± 5.0 (44)	4.0 ± 2.2 (17)	7.9 ± 4.2 (17)	10q(haplo-type-1)	16.6 ± 2.0 (24)	11.8 ± 2.5 (20)	17.6 ± 2.8 (19)
10p	8.6 ± 4.2 (39)	4.6 ± 2.1 (28)	10.4 ± 3.6 (42)	10q(haplo-type-2)	no haplotype-2	no haplotype-2	9.7 ± 3.9 (12)
11p*	15.6 ± 4.5 (5)	12.0 ± 1.4 (13)	17.5 ± 2.9 (10)	10q(haplo-type-3)	4.8 ± 1.8 (16)	3.2 ± 2.2 (7)	no haplotype-3
12p	8.9 ± 6.4 (18)	2.3 ± 1.9 (13)	11.8 ± 5.8 (22)	11q	9.7 ± 4.6 (78)	4.1 ± 2.0 (36)	10.6 ± 5.2 (54)
13p	nd	nd	nd	12q	7.6 ± 3.2 (77)	2.7 ± 1.6 (29)	9.8 ± 4.5 (79)
14p	nd	nd	nd	13q	9.6 ± 5.1 (67)	3.8 ± 1.4 (36)	11.7± 7.2 (32)
15p	nd	nd	nd	14q	9.3 ± 4.7 (39)	4.9 ± 2.3 (16)	8.8 ± 3.5 (15)
16p	nd	nd	nd	15q(haplo-type-1)*	19.5 ± 9.5 (33)	nd	15.1 ± 5.4 (18)
17p	nd	nd	nd	15q(haplo-type-2)	15.0 ±5.5 (21)	4.9 ± 2.1 (20)	10.2 ± 5.5 (18)
18p	9.6 ± 6.6 (42)	4.4 ± 1.6 (15)	13.0 ± 5.2 (28)	16q(haplo-type-1)*	7.8 ± 3.1 (77)	6.0 ± 1.7 (28)	7.0 ± 3.5 (28)
19p	7.1 ± 3.8 (121)	3.7 ± 2.6 (32)	10.1 ± 4.9 (23)	16q(haplo-type-2)	6.1 ± 2.7 (39)	1.5 ± 1.1 (17)	9.1 ± 5.8 (10)
20p*	17.0 ± 3.2 (23)	6.0 ± 2.0 (10)	13.1 ± 5.5 (7)	17q	7.1 ± 2.9 (43)	5.6 ± 4.4 (20)	10.9 ± 3.6 (23)
21p	nd	nd	nd	18q	9.3 ± 4.4 (43)	3.3 ± 3.7 (36)	9.6 ± 5.1 (36)
22p	nd	nd	nd	19q	nd	nd	nd
Xp/Yp	nd	nd	nd	20q	2.2 ± 1.9 (64)	13.4 ± 2.0 (23)	3.5 ± 1.3 (17)
				21q	8.9 ± 3.8 (70)	5.2 ± 3.6 (25)	8.8 ± 6.6 (47)
				22q	nd	nd	nd
				Xq/Yq	7.9 ± 4.3 (14)	2.6 ± 1.6 (15)	8.5 ± 3.4 (20)

* = Gap-filling alleles and structurally variant haplotypes characterized only by single-molecule mapping data.

Differences in telomere lengths of different haplotypes are observed. The 10q telomere of NA12878 has 16.6 kb telomere length for haplotype 1, whereas the other NA12878 haplotype of 10q possesses only 4.8 kb telomere length. The 10q of samples NA12891 and NA12892 also show the telomere length differences between haplotypes. On the other hand, the 16q of NA12878 shows little difference in telomere length between the two haplotypes. While haplotype-dependent telomere length differences have been observed previously (5–7,35), the effectiveness and throughput of this single molecule measurement technique in distinguishing telomere length haplotypes globally provides proof-of-principle for the method and demonstrates its potential for routine tracking of this rarely-investigated modality of telomere biology.

NA12891 has the shortest telomeres for almost every chromosome among the trio, reflecting shorter average telomere length genome-wide for this lymphoblastoid cell line relative to the other two. Lymphoblastoid cell line differences in average telomere length likely reflect differences in the extent of lymphocyte clonal proliferation prior to Epstein-Barr Virus (EBV) mediated immortalization as well as other unknown factors in the EBV transformation process; cell line-specific differences are not necessarily expected to reflect the normal telomere lengths in the progenitor lymphocytes used for immortalization. Similar telomere measurements of DNA from leukocyte populations derived directly from blood samples are required for telomere length heritability studies that will combine allele-specific telomere length measurements with linked long-range haplotypes.

Since our telomere length estimate is based on mapping single molecules back to the reference sequence, knowing the complete reference sequence from the centromeric end of the (TTAGGG)n tract through the subtelomere repeats into 1-copy DNA is important. For the telomeres with gaps and structurally variant haplotypes characterized here only through single-molecule mapping (Table 2, telomeres with asterisks), the exact subtelomeric start site for the (TTAGGG)n tract on the mapped molecules is unknown relative to the most distal recognition site for the Nicking enzyme Nt.BspQI. This site occurs on average every 9.3 kb in the human genome, so the (TTAGGG)n lengths at these telomeres as measured by this single-molecule method will be overestimated (explaining their generally longer than average estimated (TTAGGG)n tract lengths (Table 2)). This mapping method for telomere length measurement may also slightly overestimate some telomere lengths due to hypervariable telomere-like sequences sometimes adjacent to (TTAGGG)n tracts (37). However, even with these caveats, this method is uniquely powerful for analyzing haplotype-resolved changes in telomere length (delta TL) that occur within individual genomes. For example, longitudinal studies of telomere lengths in individuals are amenable to this approach, and are expected to reveal haplotype-resolved age and environment-associated telomere delta TL over time and exposures, respectively. Similarly, the method will enable experimental studies of haplotype-resolved delta TL as they relate to biology and genetics in cultured cell lines, including detailed analyses of cis-effects on delta TL at individual telomeres. Ultimately, optimization of a new CRISPR/Cas9 directed telomere labeling protocol (38) may be combined with our method to provide a more direct readout of telomere length on these single molecules, removing this potential limitation entirely.

## DISCUSSION

In this study, we applied a highly automated single-molecule mapping technology in nano-channel arrays (24,25) to use high molecular weight genomic DNA molecules for *de novo* map assembly in six human genomes: a CEPH trio (NA12878, NA12891 and NA12892) and three additional unrelated genomes HG02490, HG02603 and HG02522. (Table 1). We prepared long DNA molecules and collected single-molecule data (>150 kb) to a minimum of 71× depth of coverage for each individual. The assembled contigs were compared and aligned separately with either human reference genome hg38 or with updated subtelomere reference assemblies (23). A total of 36 of 46 subtelomeric regions were successfully mapped and analyzed. The 13p, 14p, 15p, 21p and 22p subtelomeres were not studied due to the lack of reference sequences for these acrocentric short-arm telomeres. Two of six genomes have continuous Xp/Yp contigs, but none covered the 200 kb telomere-adjacent sequences of the XpYp subtelomere reference, possibly due to the many gaps in available reference sequences near this subtelomere. Inverted nick pair (INP) sites, where two closely-spaced nicking enzyme sites (in this case Nt.BspQI sites) are found on opposite strands of the DNA and thus cause double-strand breaks in molecules to be mapped, precluded the assembly of long continuous contigs for 16p, 17p, 19q and 22q. Additional data sets with different nicking-enzymes are required to bridge these INP gaps in single-molecule maps.

Particular strengths of this mapping method are its ability to precisely delineate genomic sequence gaps, detect and track new SVs and connect individual duplicated genome segments and SVs with adjacent long-range haplotypes. Each of these strengths were demonstrated in our analysis of these six genomes, revealing new information on human subtelomere gap size and features, structural variations and long-range subtelomere haplotypes. These features make the method a uniquely valuable new tool for improving the quality of genome assemblies in complex DNA regions, and especially important for incorporating next-gen sequence data for these regions into current genome assemblies. While particularly valuable for human subtelomeric regions, where the number and organization of SREs can contribute to very large polymorphisms and alternative haplotypes for single telomeres (38–45), these methods can find wide application for improving the assembly quality of complex regions of any genome.

Telomere length estimates by single-molecule genome mapping, as described here, have the potential to link allele-specific single telomere lengths to SRE organization and long-range haplotypes. Even with the current caveats to this method, it is already uniquely amenable to haplotype-resolved studies of delta TL within individual genomes over time or in response to environmental exposures and/or experimental manipulations. This may provide, for the first time, a tractable method for mechanistically deciphering long-range cis effects on telomere length regulation and stability. Future improvements incorporating direct TL detection strategies into single molecule mapping (38) may broaden the applicability to haplotype-resolved TL comparisons between genomes.

## Supplementary Material

Supplementary Data
