# Supplementary material for: High-throughput single-molecule mapping links subtelomeric variants and long-range haplotypes with specific telomeres
Source: Nucleic Acids Res. 2017 Feb 9;45(9):e73. doi: 10.1093/nar/gkx017 (PMC5605236; doi:10.1093/nar/gkx017)

Alignment of single-molecule mapping data to subtelomeres of hg38 and the Stong et al (2014) assemblies.

The two subtelomere assemblies to which the single-molecule data were mapped differ in several important respects, summarized in Supplementary Table S1. All telomere-terminal genomic regions are represented in the current public human reference sequence assembly (hg38, December 2013) as a 10 kb gap (based upon the estimated average size of unsequenced terminal (TTAGGG)n tracts) at the beginning and the end of each chromosomal assembly. At some euchromatic telomeres, clone gaps exist adjacent to the (TTAGGG)n tracts, and these are represented as additional gaps adjacent to the 10 kb telomere gap in hg38. In the special case of the heterochromatic short-arm telomeres (13p, 14p, 15p, 21p, 22p), multi-megabase-sized gaps representative of the p-arm regions of the acrocentric chromosomes and their adjacent centromeres are designated, and preclude the use of reference sequence data from the terminal regions of these chromosome arms. Stong et al. (2014) developed updated subtelomere assemblies and modified their representation in order to facilitate their global analysis. They removed distal telomere (TTAGGG)n sequences and distal gaps, so that coordinate 1 of each complete assembly corresponded to the start of the terminal repeat tract on the strand oriented towards the centromere. This maintained a consistent starting coordinate for subtelomere annotation and permitted direct comparisons of p-arm and q-arm subtelomere sequence organization (Stong et al., 2014). For subtelomeres with telomere-adjacent gaps, Stong et al. designated coordinate 1 the most distal base of available subtelomere sequence in that assembly.

Alignment of single-molecule mapping data to the Stong et al assemblies simply matches the single-molecule maps with in silico maps of nicking sites in each assembly. Depending upon the specific subtelomere, molecules extending beyond the assemblies can fill gap regions, represent structural variants relative to the reference, and/or be used to measure specific (TTAGGG)n tract lengths. Alignment to hg38 subtelomeres is similar in principle, but the representations of the reference sequence include segments of presumptive gap DNA completely lacking in silico nicking sites (the gaps are represented by strings of NNN’s in hg38; see supplementary figures S1 to S5). Here, aligned single molecules can either end near the start of the designated gap regions, or extend into or through them, depending on the characteristics of the source subtelomeric DNA and the sizes of the presumptive gaps represented in hg38. Measurement of (TTAGGG)n tracts relative to hg38 were calculated in the same manner as for the Stong et al (2014) alignments, and depend only upon the distance between the most distal nicking site and the start of the (TTAGGG)n tract in the respective reference sequence (see Figure 5). Additional differences between the assemblies at specific subtelomeres are noted in Supplementary Table S1 and have been described previously (Stong et al., 2014).

Supplementary Table S1. Human telomere-adjacent gaps designated in hg38 and in Stong et al. (2014).

Tel	hg38 Gap size (kb)	Stong et al., 2014<br>G = telomere adjacent gap<br>T = no gap	Comments
1p	586*	G	The distal 586 kb of the hg38 assembly consists of several concatenated alternative subtelomere alleles and is not useful for alignment to mapped long DNA molecules.
1q	10	T	
2p	10	T	
2q	10	T	
3p	10	T	
3q	60	G	
4p	10	T	
4q	10	T	hg38 also designates a 50 kb gap within the D4Z4 repeat array in 4q
5p	10	T	
5q	60	T	
6p	60	G	
6q	60	T	
7p	10	T	
7q	10	T	
8p	60	G	
8q	60	T	
9p	10	T	
9q	60	G	
10p	10	T	
10q	10	T	hg38 also designates a 50 kb gap within the D4Z4 repeat array in 10q
11p	60	G	
11q	10	T	
12p	10	T	
12q	10	T	
13p	16,000	nd	p-arm telomere
13q	10	T	
14p	16,000	nd	p-arm telomere
14q	160	T	Alternative 14q subtelomere haplotypes are represented in the two assemblies
15p	17,000	nd	p-arm telomere
15q	10	T	
16p	10	T	
16q	110	T	
17p	60	T	
17q	10	T	
18p	10	T	

18q	110	T	
19p	60	T	
19q	10	T	
20p	60	G	
20q	110	T	
21p	5,000	nd	p-arm telomere
21q	10	T	
22p	10,500	nd	p-arm telomere
22q	10	T	
Xp/Yp	10	T	
Xq/Yq	10	T	

nd = no data

Stong N, Deng Z, Gupta R, Hu S, Paul S, Weiner AK, Eichler EE, Graves T, Fronick CC, Courtney L et al. 2014. Subtelomeric CTCF and cohesin binding site organization using improved subtelomere assemblies and a novel annotation pipeline. *Genome Research* **24**(6): 1039-1050.

Supplementary Table S2. Structural variants.

Chr	Sample	Ref Start	Ref End	size (bp)	Type
1q	HG02522	248518579	248539532	5551.2	deletion
1q	HG02603	248518579	248530244	5912.7	deletion
1q	HG02490	248518579	248539532	5566.8	deletion
2p	HG02490	202934	210503	4582.2	insertion
2p	HG02603	209508	210503	17671	insertion
2q	HG02490	241887084	241897354	5799	insertion
2q	HG02522	242004872	242012317	11866.9	insertion
2q	HG02603	241885992	241897354	8884.3	insertion
4p	HG02490	126300	147992	6251	deletion
4p	HG02522	126300	147992	6178.2	deletion
7p	HG02522	149009	158080	9071	deletion
7p	HG02522	209809	219755	2213.5	insertion
7q	HG02490	159194140	159205819	5069.3	insertion
7q	HG02603	159194140	159205819	4563.2	insertion
7q	HG02522	158909681	158922450	8232.3	insertion
7q	HG02603	158891281	158922450	4880.8	insertion
7q	HG02490	158891281	158922450	6835.4	insertion
10q	HG02522	133658071	133767255	5223.5	deletion
10q	HG02603	133658071	133767255	66562.8	deletion
10q	HG02490	133658071	133767255	46107.9	deletion
11p	HG02490	174006	184906	5652	insertion
11p	HG02522	171877	184906	6800.9	insertion
11p	HG02490	336372	366388	13919	insertion
14q	HG02490	106698803	106712361	2134	insertion
14q	HG02522	106698803	106745274	60962	deletions
14q	HG02603	106698803	106745274	46471	deletions
14q	HG02490	106745274	106759765	4292	insertion
15q	HG02490	101537059	101547744	2516	insertion
15q	HG02603	101537059	101547744	15361	insertion
15q	HG02490	101747832	101769186	9080	insertion
15q	HG02522	101747832	101769186	12262	insertion
15q	HG02603	101747832	101769186	20182	insertion
15q	HG02490	101883941	101906637	3300	insertion
15q	HG02522	101883941	101906637	5994	insertion
17p	HG02490	260233.5	353172	7068.2	insertion
17p	HG02603	299520	354584	14042.2	insertion
17p	HG02522	299520	330238	10382.7	insertion
17p	HG02603	441139	506071	39435.4	deletion
17p	HG02522	441139	506071	39236.9	deletion
17p	HG02490	441139	506071	40103.1	deletion
19p	HG02490	423961	434633	5450	insertion
19p	HG02603	423961	434633	3919	insertion

Supplementary Figure legends:

Figure S1. Consensus single-molecule maps of individual subtelomeres 3q, 7p, 11p, 9q and 20q aligned with the hg38 reference. Consensus single-molecule maps (yellow lines) from the designated individual genomes (on top of each reference sequence panel) were aligned to individual subtelomeres in the hg38 reference (blue bars with vertical black lines across the bars). The consensus maps complete the telomere adjacent DNA (red double-arrow lines) for each of these respective subtelomeres, where hg38 indicates gaps of the approximate size shown (gray arrows).

Figure S2. Consensus single-molecule maps of the 16q and 20p telomeres aligned with the hg38 reference. Panel A: Consensus maps from the NA12892, NA12891, NA12878 and HG02490 genomes each show allele(s) lacking a large gap at 16q, whereas consensus maps from HG02522 and HG02603 both show allele(s) containing additional subtelomeric DNA sufficient to fill the large gap designated in hg38 (gray arrows). Panel B: Consensus maps of 20p for the six genomes reveal three distinct structural variants (red arrows), each of which contains subtelomeric DNA extending beyond the gap designated in hg38 (gray arrows).

Figure S3. Comparison of 14q single-molecule consensus maps among different genomes. The polymorphism of 14q consensus maps consists of multiple insertions (green bars) and deletions (orange bars) between different individuals. None of the alleles from these genomes appear to extend into the large telomere-adjacent gap region (gray arrows) designated at 14q in hg38.

Figure S4. Consensus single-molecule maps reveal both a deletion (orange bar) and variable sized tracts of the macrosatellite D4Z4 repeat (red arrows) in the 4q subtelomere. The hg38 reference includes a 10 kb gap designating (TTAGGG)n sequences (gray arrow at telomere) and a 50 kb gap as part of the D4Z4 repeat array within the macrosatellite region. There are no nicking sites within the D4Z4 repeat array.

Figure S5. Comparison of 10q single-molecule consensus maps among different genomes. The hg38 reference includes a 10 kb gap designating (TTAGGG)n sequences (gray arrow at telomere) and a 50 kb gap as part of the D4Z4 repeat array (gray arrows). There are no nicking sites within the D4Z4 repeat array. Four distinct 10q haplotypes, differing in D4Z4 array size (red arrows)can be detected, two of them in HG02603. The 10q consensus maps of related genomes NA12878, NA12891 and NA12892 also have a large 139kb insertion (green bar) 400kb upstream from the telomere.

Figure S1. Consensus maps of 3q, 7p, 11p, 9q and 20q vs. the reference.

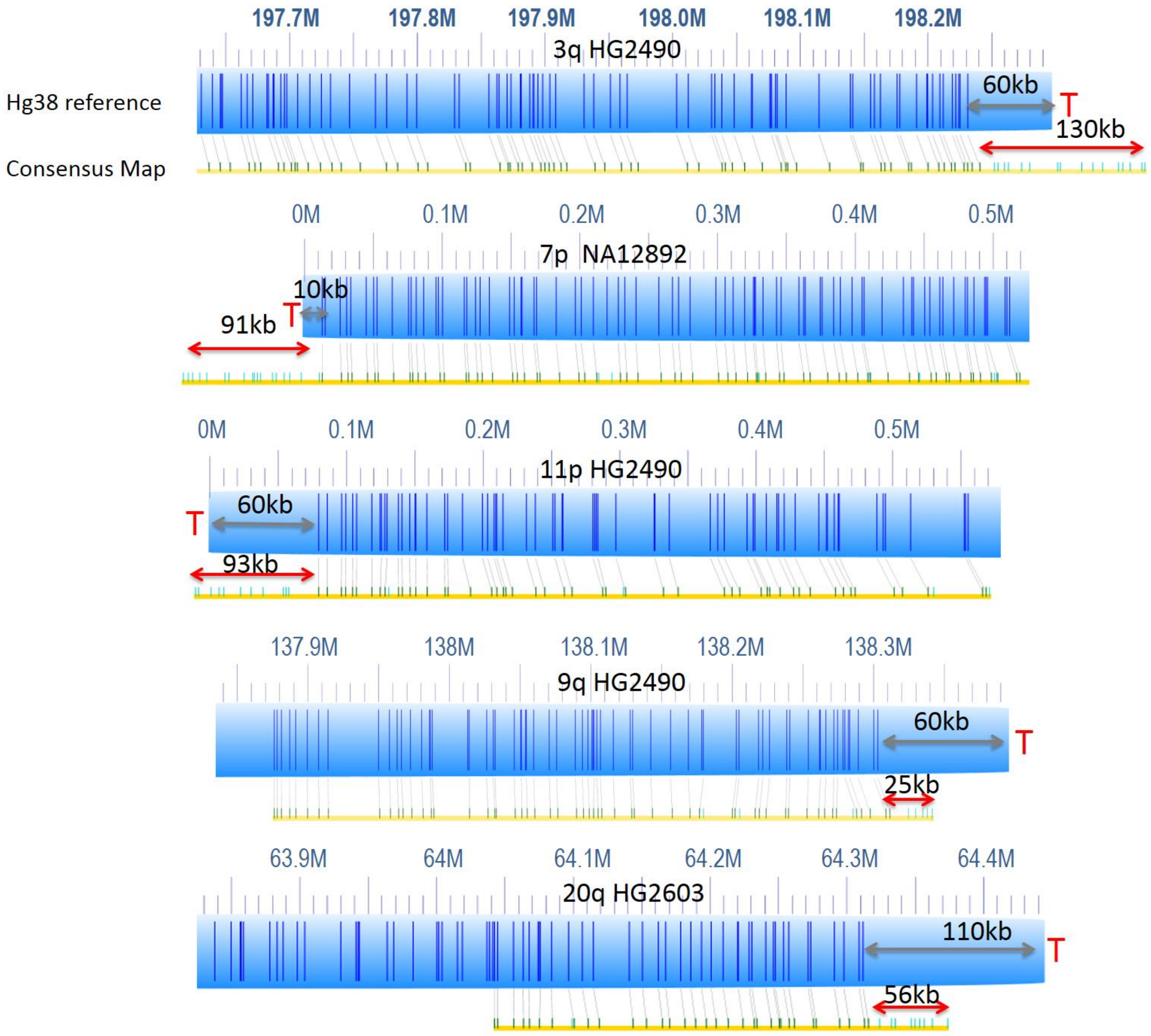

S2. Consensus maps of 16q and 20p vs. the reference.

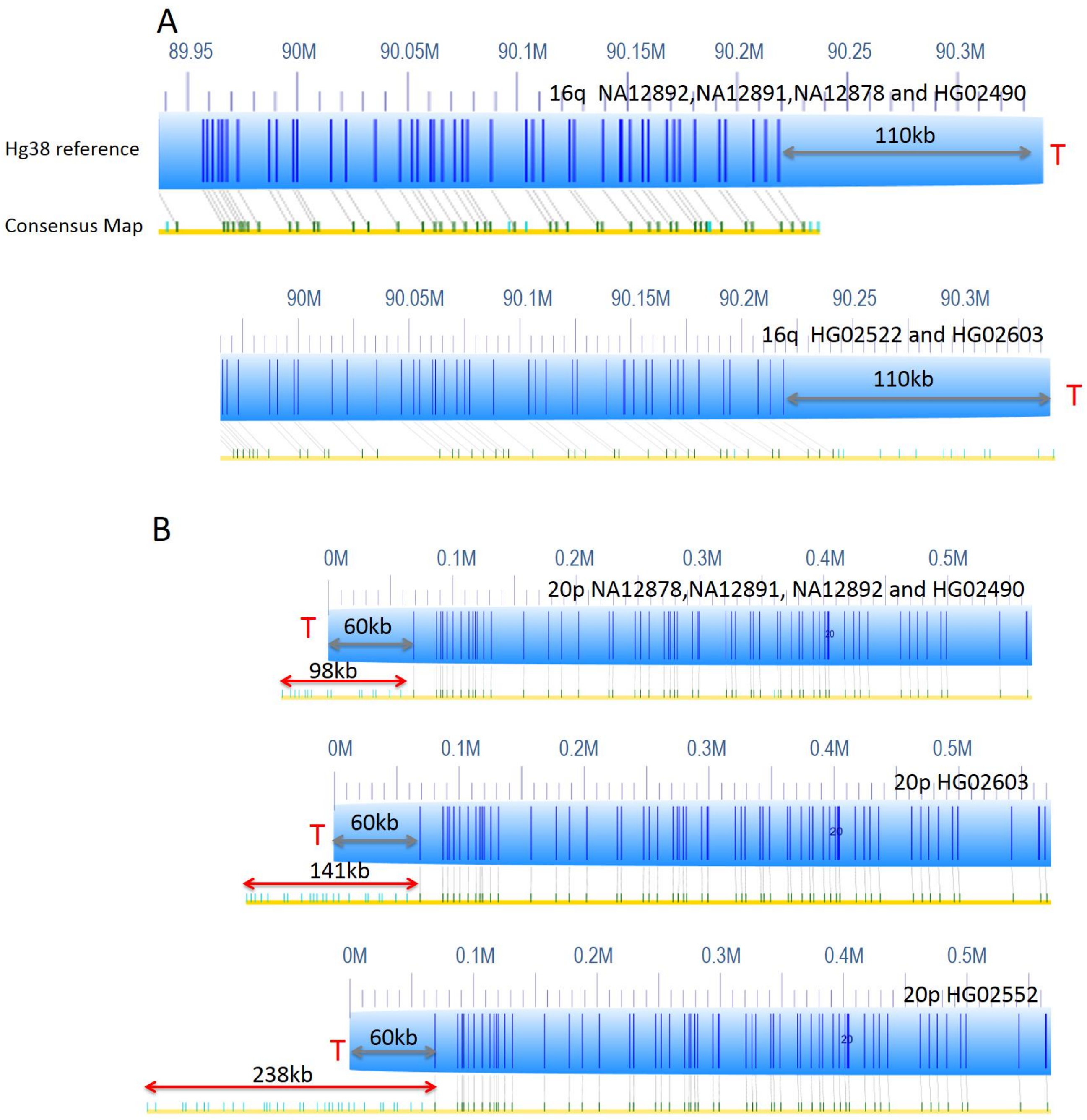

S3. 14q consensus map comparison between different genomes.

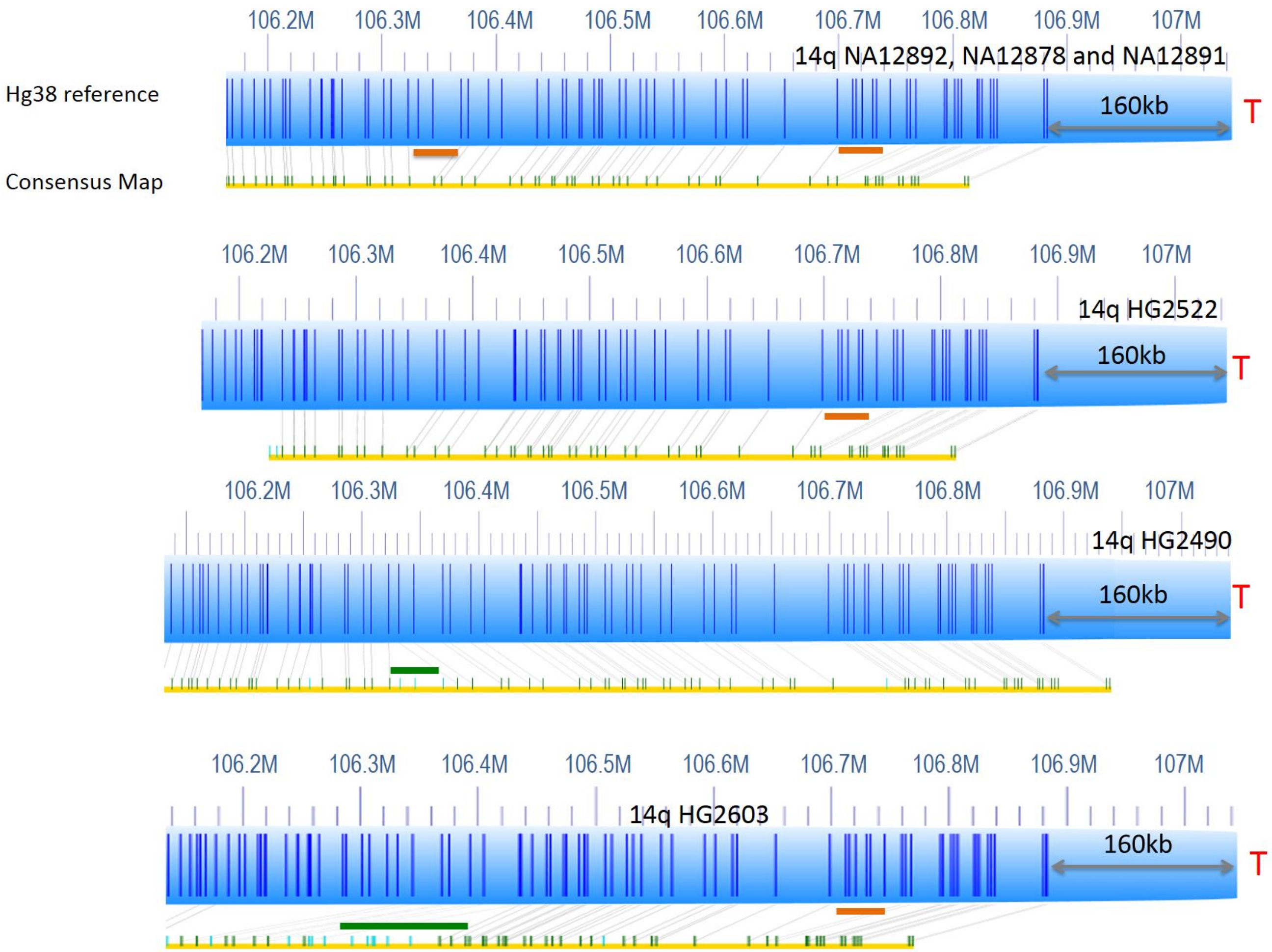

S4. 4q consensus map comparison between different genomes.

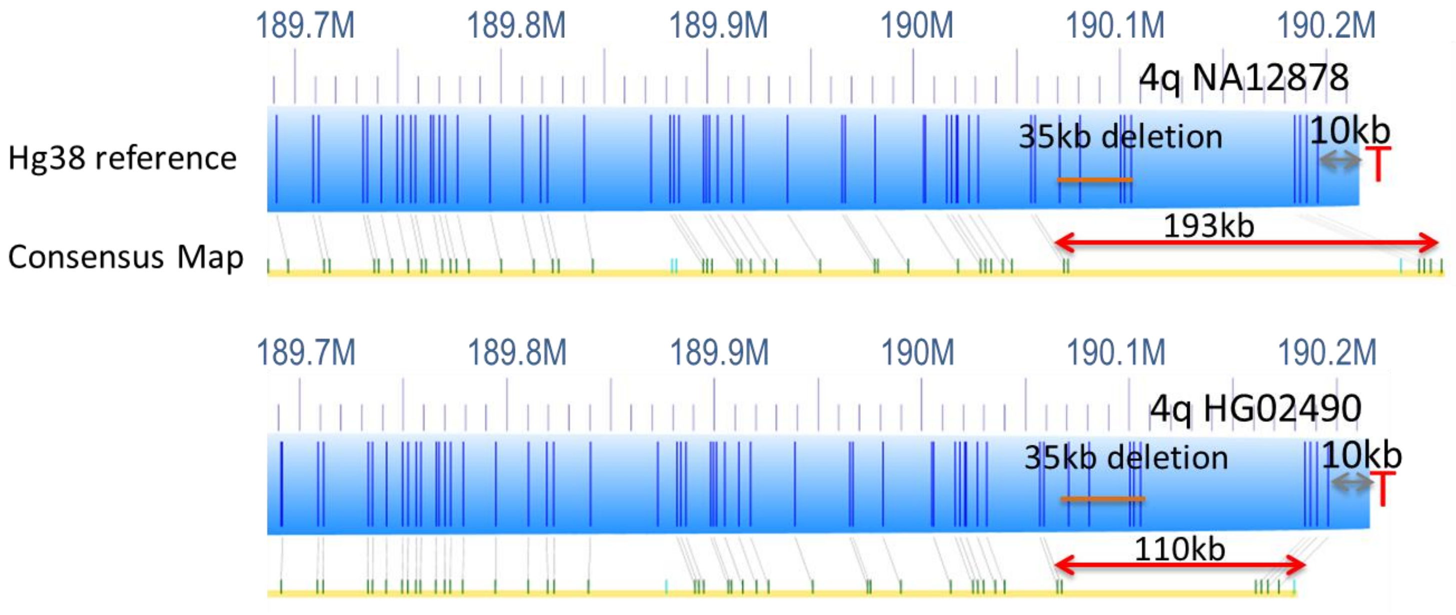

S5. Consensus maps of 10q vs. the reference

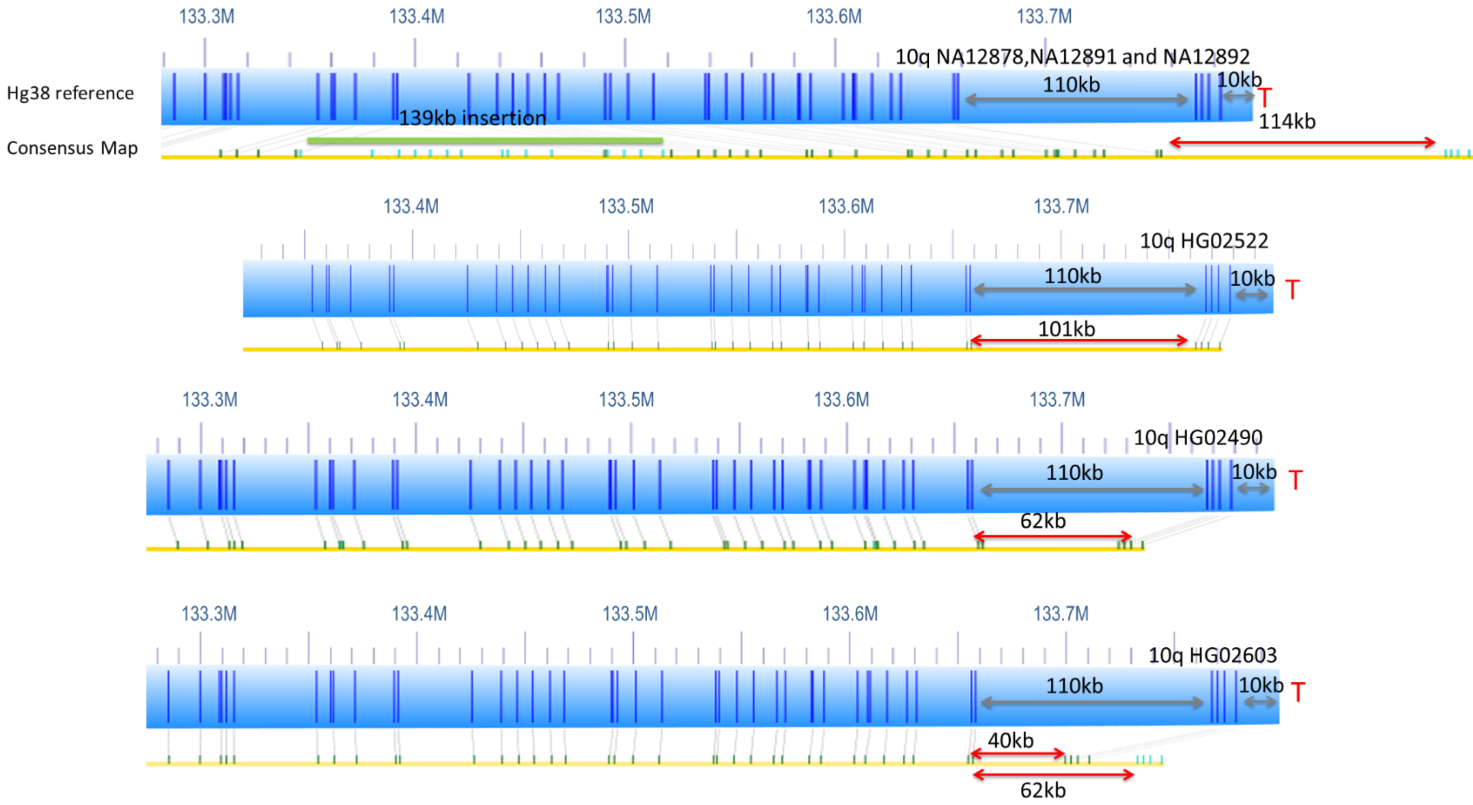

Supplement: Supplementary Data [file gkx017_supp.pdf]
